# Transcriptomic analysis of the late stages of grapevine (*Vitis vinifera* cv. Cabernet Sauvignon) berry ripening reveals significant induction of ethylene signaling and flavor pathways in the skin

**DOI:** 10.1186/s12870-014-0370-8

**Published:** 2014-12-19

**Authors:** Grant R Cramer, Ryan Ghan, Karen A Schlauch, Richard L Tillett, Hildegarde Heymann, Alberto Ferrarini, Massimo Delledonne, Sara Zenoni, Marianna Fasoli, Mario Pezzotti

**Affiliations:** Department of Biochemistry and Molecular Biology, University of Nevada, Reno, NV 89557 USA; Department of Viticulture and Enology, University of California, Davis, CA 95616 USA; Department of Biotechnology, University of Verona, Strada Le Grazie 15, I-37134 Verona, Italy; Center for Bioinformatics, University of Nevada, Reno, NV 89557 USA

**Keywords:** Ethylene, Fruit ripening, Grape, Microarray, *Vitis vinifera* L

## Abstract

**Background:**

Grapevine berry, a nonclimacteric fruit, has three developmental stages; the last one is when berry color and sugar increase. Flavors derived from terpenoid and fatty acid metabolism develop at the very end of this ripening stage. The transcriptomic response of pulp and skin of Cabernet Sauvignon berries in the late stages of ripening between 22 and 37 °Brix was assessed using whole-genome micorarrays.

**Results:**

The transcript abundance of approximately 18,000 genes changed with °Brix and tissue type. There were a large number of changes in many gene ontology (GO) categories involving metabolism, signaling and abiotic stress. GO categories reflecting tissue differences were overrepresented in photosynthesis, isoprenoid metabolism and pigment biosynthesis. Detailed analysis of the interaction of the skin and pulp with °Brix revealed that there were statistically significantly higher abundances of transcripts changing with °Brix in the skin that were involved in ethylene signaling, isoprenoid and fatty acid metabolism. Many transcripts were peaking around known optimal fruit stages for flavor production. The transcript abundance of approximately two-thirds of the AP2/ERF superfamily of transcription factors changed during these developmental stages. The transcript abundance of a unique clade of ERF6-type transcription factors had the largest changes in the skin and clustered with genes involved in ethylene, senescence, and fruit flavor production including ACC oxidase, terpene synthases, and lipoxygenases. The transcript abundance of important transcription factors involved in fruit ripening was also higher in the skin.

**Conclusions:**

A detailed analysis of the transcriptome dynamics during late stages of ripening of grapevine berries revealed that these berries went through massive transcriptional changes in gene ontology categories involving chemical signaling and metabolism in both the pulp and skin, particularly in the skin. Changes in the transcript abundance of genes involved in the ethylene signaling pathway of this nonclimacteric fruit were statistically significant in the late stages of ripening when the production of transcripts for important flavor and aroma compounds were at their highest. Ethylene transcription factors known to play a role in leaf senescence also appear to play a role in fruit senescence. Ethylene may play a bigger role than previously thought in this non-climacteric fruit.

**Electronic supplementary material:**

The online version of this article (doi:10.1186/s12870-014-0370-8) contains supplementary material, which is available to authorized users.

## Background

Grapevine berry ripening can be divided into three major stages [1]. In stage 1, berry size increases sigmoidally. Stage 2 is known as a lag phase where there is no increase in berry size. Stage 3 is considered the ripening stage. Veraison is at the beginning of the ripening stage and is characterized by the initiation of color development, softening of the berry and rapid accumulation of the hexoses, glucose and fructose. Berry growth is sigmoidal in Stage 3 and the berries double in size. Many of the flavor compounds and volatile aromas are derived from the skin and synthesized at the end of this stage [2-4].

Many grape flavor compounds are produced as glycosylated, cysteinylated and glutathionylated precursors (e.g. terpenoids and C_13_-norisoprenoids) and phenolics [3,5-8] and many of the precursors of the flavor compounds are converted to various flavors by yeast during the fermentation process of wine. Nevertheless, there are distinct fruit flavors and aromas that are produced and can be tasted in the fruit, many of which are derived from terpenoids, fatty acids and amino acids [3,7,9-13].

Terpenes are important compounds for distinguishing important cultivar fruit characteristics [11,12]. There are 69 putatively functional, 20 partial and 63 partial pseudogenes in the terpene synthase family that have been identified in the Pinot Noir reference genome [12]. Terpene synthases are multi-functional enzymes using multiple substrates and producing multiple products. More than half of the putatively functional terpene synthases in the Pinot Noir reference genome have been functionally annotated experimentally and distinct differences have been found in some of these enzymes amongst three grape varieties: Pinot Noir, Cabernet Sauvignon and Gewürztraminer [12].

Other aromatic compounds also contribute significant cultivar characteristics. C_13_-norisoprenoids are flavor compounds derived from carotenoids by the action of the carotenoid cleavage dioxygenase enzymes (CCDs) [11]. Cabernet Sauvignon, Sauvignon Blanc and Cabernet Franc are characterized by specific volatile thiols [14,15] and methoxypyrazines [16-18]. Enzymes involved in the production of these aromas have been recently characterized [8,19].

Phenolic compounds play a central role in the physical mouthfeel properties of red wine; recent work relates quality with tannin levels [20,21]. While the grape genotype has a tremendous impact on tannin content, the environment also plays a very large role in grape composition [22]. The pathway for phenolic biosynthesis is well known, but the mechanisms of environmental influence are poorly understood.

Ultimately, there is an interaction between molecular genetics and the environment. Flavor is influenced by climate, topography and viticultural practices (i.e. irrigation, canopy management, etc.) [23]. For example, water deficit alters gene expression of enzymes involved in aroma biosynthesis in grapes, which is genotype dependent, and may lead to increased levels of compounds, such as terpenes and hexyl acetate, that contribute to fruity volatile aromas [24,25].

The grapevine berry can be subdivided into the skin, pulp and seeds [26]. The skin includes the outer epidermis (single cell layer) and inner hypodermis (from 1 to 17 cell layers). A thick waxy cuticle covers the epidermis. The hypodermal cells contain chloroplasts, which lose their chlorophyll at veraison and become modified plastids [27]; they are the sites of terpenoid biosynthesis and carotenoid catabolism. Anthocyanins and tannins accumulate in the vacuoles of hypodermal cells [2]. Pulp cells are the main contributors to the sugar and organic acid content of the berries [2]. Pulp cells also have a much higher set of transcripts involved in carbohydrate metabolism, but a lower set of transcripts involved in lipid, amino acid, vitamin, nitrogen and sulfur metabolism than in the skins [4].

Hormones can influence berry development and ripening. Concentrations of auxin, cytokinins and gibberellins tend to increase in early fruit development of the first stage [1]. At veraison, these hormone concentrations have declined concomitant with a peak in abscisic acid concentration just before veraison. Auxin prolongs the Stage 2 lag phase [28] and inhibits anthocyanin biosynthesis and color development in Stage 3 [29].

Grapevine, a nonclimacteric fruit, is not very sensitive to ethylene; however, ethylene appears to be necessary for normal fruit ripening [30-32]. Ethylene concentration is highest at anthesis, but declines to low levels upon fruit set; ethylene concentrations rise slightly thereafter and peak just before veraison then decline to low levels by maturity [33]. Ethylene also plays a role in the ripening of another nonclimacteric fruit, strawberry [34,35]. ABA also appears to be important in grape berry ripening during veraison when ABA concentrations increase resulting in increased expression of anthocyanin biosynthetic genes and anthocyanin accumulation in the skin [24,29,36-38]. ABA induces ABF2, a transcription factor (TF) that affects berry ripening by stimulating berry softening and phenylpropanoid accumulation [39]. In addition, ABA affects sugar accumulation in ripening berries by stimulating acid invertase activity [40] and the induction of sugar transporters [41,42]. It is not clear whether ABA directly affects flavor volatiles (C_13_-norisoprenoids), but there could be indirect effects due to competition for common precursors in the carotenoid pathway.

Many grape berry ripening studies have focused on targeted sampling over a broad range of berry development stages, but generally with an emphasis around veraison, when berry ripening is considered to begin. In this study, a narrower focus is taken on the late ripening stages where many berry flavors are known to develop in the skin. We show that that the abundance of transcripts involved in ethylene signaling is increased along with those associated with terpenoid and fatty acid metabolism, particularly in the skin.

## Results

### The transcript abundance of a large number of genes was statistically significantly changed across °Brix levels and berry tissues

Cabernet Sauvignon clusters were harvested in 2008 from a commercial vineyard in Paso Robles, California at various times after veraison with a focus on targeting °Brix levels near maturity. Dates and metabolic details that establish the developmental state of the berries at each harvest are presented in Additional file 1. Berries advanced by harvest date with the typical developmental changes for Cabernet Sauvignon: decreases in titratable acidity and 2-isobutyl-3-methoxypyrazine (IBMP) concentrations and increases in sugar (°Brix) and color (anthocyanins).

Transcriptomic analysis focused on four harvest dates having average cluster °Brix levels of 22.6, 23.2, 25.0 and 36.7. Wines made in an earlier study from grapes harvested at comparable levels of sugars or total soluble solids to those in the present study showed clear sensory differences [43]. Six biological replicates, comprising two clusters each, were separated into skins and pulp in preparation for RNA extraction and transcriptomic analysis using the NimbleGen Grape Whole-Genome Microarray. Thus, a 4 × 2 factorial (°Brix x Tissue) experimental design was established. After standard microarray processing and data normalization, two-way ANOVA indicated that the transcript abundance of 16,280 transcripts statistically significantly changed across the °Brix levels below the adjusted p-value (upon a correction for the false discovery rate [44]) of 0.05 (herein referred to as “significant” throughout this paper), the transcript abundance of 10,581 transcripts changed significantly across Tissue types, and the abundance of 2053 transcripts changed significantly with respect to the °Brix x Tissue interaction term (Additional file 2, to view these transcripts, sort from lowest to highest in the appropriate adjusted (adj) p-value column: adjBrix, adjTissue or adjTissue*Brix).

A note of caution must be added here. There are high similarities amongst members in certain *Vitis* gene families (e.g. ERF TFs, stilbene and terpene synthases), making it very likely that cross-hybridization can occur with probes on the microarray with high similarity to other genes. We estimate approximately 13,000 genes have the potential for cross-hybridization, with at least one probe of a set of four unique probes for that gene on the microarray potentially cross-hybridizing with probes for another gene on the microarray. Genes with the potential for cross-hybridization have been identified and are highlighted in light red in Additional file 2. The rationale to include them is that although individual genes can not be uniquely separated, the probe sets can identify a gene and its highly similar gene family members, thus, providing some useful information about the biological responses of the plant. An additional approach was taken, removing cross-hybridizing probes before quantitative data analysis (data not shown). Many of the significant genes were unaffected by this processing, but 3600 genes (e.g. many terpene synthases and stilbene synthases) were completely removed from the analysis. Thus, it was felt that valuable information was lost using such a stringent approach. The less stringent approach allowing for analysis of genes with potential cross-hybridization was used here in the rest of the analyses.

**Figure 1:**
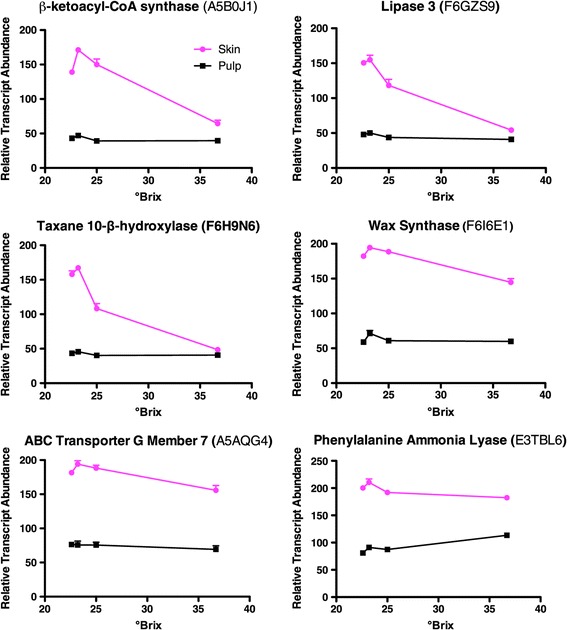
**Some representative examples of transcripts with higher abundance in the skin compared to the pulp at 23.2 °Brix.** Data are means ± SE; n = 6.

To assess the main processes affected by these treatments, the gene ontologies (GO) of significantly affected transcripts were analyzed for statistical significance (overrepresentation relative to the whole genome) using BinGO [45]. Based on transcripts that had significant changes in abundance with °Brix level, 230 biological processes were significantly overrepresented in this group (Additional file 3). The three top overrepresented processes were response to abiotic stress, biosynthetic process, and response to chemical stimulus, a rather generic set of categories. Tissue differences were more revealing at the stage when flavors peak; 4865 transcripts that were significantly higher in skins compared to pulp at 23.2 °Brix (Additional file 2) were tested for overrepresented GO functional categories (Additional file 4). Some of the top GO categories included photosynthesis, isoprenoid biosynthesis, and pigment biosynthesis (Additional file 4). Some of the transcripts with the largest differences between skin and pulp at 23.2 °Brix are β-ketoacyl-CoA synthase (fatty acid biosynthesis), taxane 10-β-hydroxylase (diterpenoid biosynthesis), wax synthase, a lipase, an ABC transporter, and phenylalanine ammonia-lyase (PAL; phenylpropanoid biosynthesis) (Figure 1).

**Figure 2:**
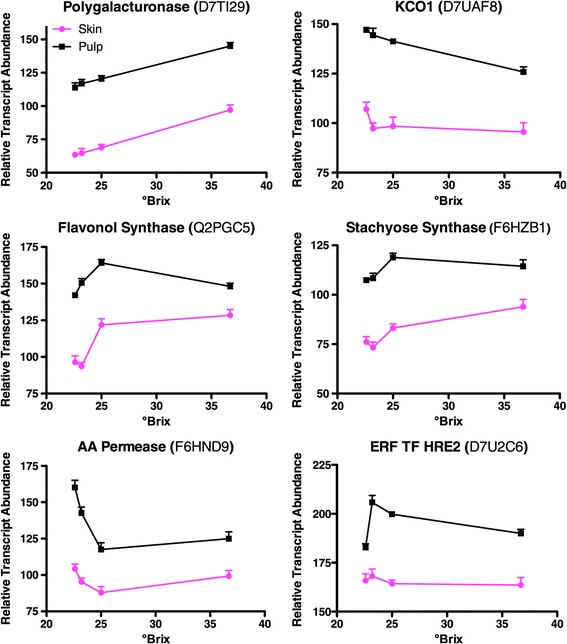
**Some representative examples of transcripts with higher abundance in the pulp compared to the skin at 23.2 °Brix.** Data are means ± SE; n = 6.

The abundance of 5716 transcripts was significantly higher in pulp than skin at 23.2 °Brix (Additional file 2). Some of the top GO categories overrepresented were a variety of transport processes (i.e. golgi-vesicle mediated transport, protein transport, ion transport, and amino acid transport) and small GTPase mediated signal transduction (Additional file 5). Some of the transcripts with the largest differences in abundance with pulp greater than skin at 23.2 °Brix were polygalacturonase (cell wall pectin degradation), flavonol synthase, stachyose synthase, an amino acid transporter, a potassium channel (KCO1), and HRE2 (hypoxia responsive ERF transcription factor) (Figure 2).

The transcript abundance of 2053 genes had significantly differential expression across °Brix levels and tissues (°Brix x Tissue interaction term). The top GO categories overrepresented in this set involved photosynthesis and phenylpropanoid metabolism, both associated with the berry skin (Additional file 6). Other flavor-centric categories of the 57 categories overrepresented include aromatic compound biosynthesis, fatty acid metabolism and alcohol catabolism.

**Figure 3:**
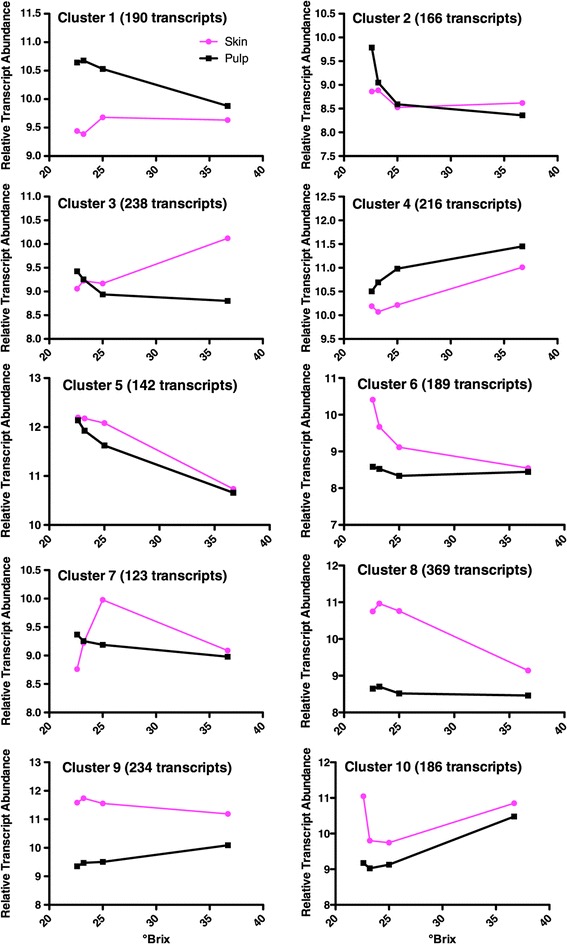
**Average profiles of all transcripts within the 10 clusters produced by k-means clustering for transcripts significantly changing with the °Brix x Tissue interaction term.**

This transcript set was further analyzed by dividing into 10 clusters using k-means clustering (Figure 3, Table 1). The overrepresented GO categories were determined for each cluster (Table 1; Additional file 7). Eight of the 10 clusters had distinct overrepresented GO categories; two clusters did not have any overrepresented GO categories, meaning that the genes in these two clusters were assigned to GO categories of expected proportions when compared to the entire NimbleGen array. Clusters 1, 8, 9 and 10 had a large number of overrepresented categories. Many GO categories within a cluster are subsets of others in that cluster and were grouped together. For example, cluster 4 had four overrepresented GO categories, oxygen transport, gas transport, heat acclimation and response to heat. The four categories could be grouped into two, as two are subsets of the others; this is how they were listed in Table 1.

**Table 1:** **Details of the 10 clusters produced by k-means clustering for the transcripts significantly changing for the °Brix x Tissue interaction term**

**Cluster #**	**# of transcripts**	**Top overrepresented GO categories**	**# of GO cat**	**Comments**
1	190	Cellular response to iron	46	Higher in pulp
		Transmembrane receptor protein tyrosine kinase signaling pathway		
2	166	Xyloglucan metabolism	11	Higher in pulp; decreasing with increasing Brix in pulp
		Growth		
3	238	Photosynthesis	12	Decreasing in pulp; increasing skin.
		Respiration		
4	216	Oxygen transport	4	Similar in both tissues; increasing with Brix
		Heat response		
5	142	None	0	Both decreasing with Brix
6	189	Amino acid phosphorylation	5	Higher in skin; decreasing in skin with Brix
7	123	None	0	Higher in skin peaking at 25 °Brix
8	369	Terpenoid metabolism	169	Higher in skin peaking at 23.2 °Brix
		Pigment biosynthesis		
		Organic acid biosynthesis		
		Amino acid phosphorylation		
		Fatty acid metabolism		
9	234	Phenylpropanoid metabolism	63	Higher in skin peaking at 23.2 °Brix
		Photosynthesis		
		Abiotic stress response		
10	186	Phenylpropanoid metabolism	26	Higher in skin decreasing at 25 °Brix
		Organic acid catabolism		

### Induction of transcripts of VviERF TFs, ethylene signaling and aroma enzymes

It would be impossible to discuss here all the transcript abundance changes detected in these berries. As we were interested in compounds associated with berry flavors as they develop or change in the late stages of berry ripening, we took a more targeted approach for analysis with this in mind. Berries at 24° Brix are known to be near-optimal for flavor [43], thus we took a simple approach to look for genes that were peaking around this stage. We found some significant and large increases in transcript abundance between the 22.6 and 23.2 °Brix levels. A group of VviERF6 transcription factor (TF) paralogs represented 6 of the top 10 transcripts increasing in transcript abundance from 22.6 to 23.2 °Brix in the skin, but not in the pulp (Additional file 2; to view, sort column O, bx23.2Skin-bx22.6Skin, from highest to lowest; this column is the ratio of values at °Brix 23.2 in the skin divided by the values °Brix 22.6 in the skin; since the values are log 2, subtracting the value in one column from the value in another column represents the ratio of the two). These VviERF6 TFs were also found in Cluster 8 (Figure 3, Table 1). This is very interesting since many flavor compounds are derived from the skin and ERF TFs are known to be responsive to ethylene, a known fruit-ripening hormone [46].

These VviERF TFs were named ERF105 in the annotation by Grimplet et al. [47] (Additional file 2), however they are more orthologous with AtERF6 as determined by a more comprehensive phylogenetic method using many plant species at Gramene (gramene.org). Annotation details of the V1 gene models of the VviAP2/ERF superfamily can be found in Additional file 8 including updated Vvi symbols according to its closest Arabidopsis ortholog as instructed by the Grapevine Gene Nomenclature System developed by the International Grape Genome Program (IGGP) Supernomenclature committee [48]. This renaming of the AP2/ERF superfamily should facilitate comparative analyses and functions with other species, particularly Arabidopsis.

To properly annotate the AP2/ERF superfamily of *Vitis vinifera* according to the IGGP Supernomenclature committee instructions, a phylogenetic tree was generated for the AP2/ERF superfamily of *Arabidopsis thaliana* and *Vitis vinifera* using the TAIR 10 and V1 gene models, respectively (Additional file 9). The labeled family classifications were derived from the *Arabidopsis* naming scheme by Nakano et al. [49]. There are 130 members in the *Vitis* AP2/ERF superfamily in the Pinot Noir reference genome. However, the six paralogs of ERF6 discussed above belong to a *Vitis vinifera* clade (12 members) in subfamily IX (31 *Vitis* members) and are distinctly different or separate from any Arabidopsis subfamily IX ERF TFs (see Unique Vitis Clade in Additional file 9). All of these TFs in this clade are orthologs of AtERF6.

**Figure 4:**
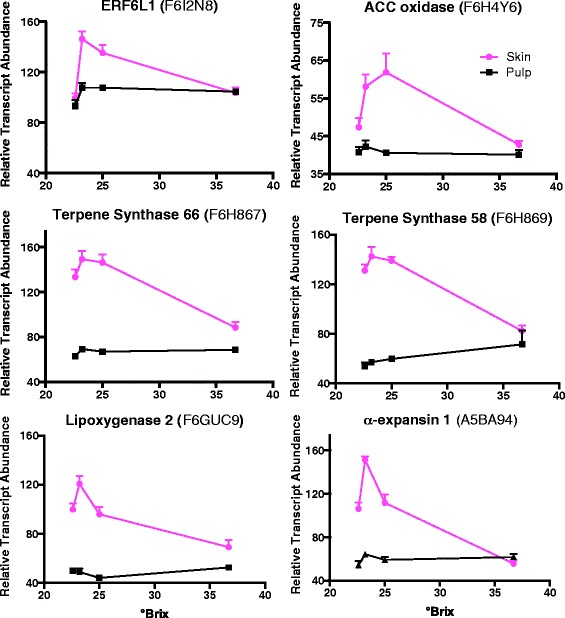
**Gene expression profiles of VviERF6L1 and 5 other transcripts in cluster 8 of the significantly changing transcripts of the °Brix x Tissue interaction set.** Data are means ± SE; n = 6.

VviERF6L1 [UniProt: F6I2N8; VIT_16s0013g00900] had one of the most interesting profiles of the 12 members of this clade because its transcript abundance peaked at 23.2 °Brix (Additional file 10). Using k-means clustering, VviERF6L1 fell within Cluster 8 (Figure 3) with 369 transcripts, including five additional VviERF6 paralogs. The top GO categories associated with Cluster 8 were genes associated with terpenoid metabolism and pigment biosynthesis (Table 1). Other interesting flavor associated categories included fatty acid and alcohol metabolism (Additional file 7). Representative transcripts from Cluster 8 that were correlated with the transcript abundance profile of VviERF6L1 can be seen in Figure 4. These are ACC oxidase, which is involved in ethylene biosynthesis; a lipoxygenase, part of a fatty acid degradation pathway giving rise to flavor alcohols such as hexenol; α-expansin 1, a cell wall loosening enzyme involved in fruit softening, and two terpene synthases, which produce important terpenes that contribute to Cabernet Sauvignon flavor and aroma. The high similarity of these transcript profiles indicates that ethylene biosynthesis and signaling may be involved in the production of grape aroma. Supporting this argument, two recent studies [50,51] have shown that a tomato ERF TF (Sl-ERF.B3), falling in the same ERF IX subfamily, has a strong effect on ethylene signaling and fruit ripening.

**Figure 5:**
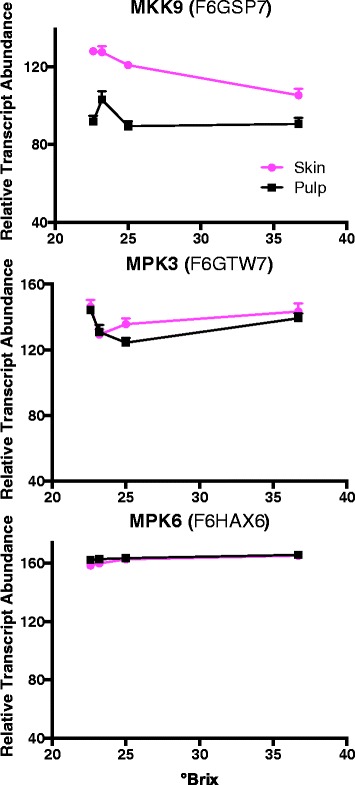
**The expression profiles of three MAP kinases.** Data are means ± SE; n = 6.

The transcript abundance of AtERF6 in Arabidopsis is strongly increased by ethylene, which is triggered by the MKK9/MPK3/MPK6 pathway [52]. The transcript abundance of VviMKK9 in the Cabernet Sauvignon berries was higher in the skin than the pulp, but there were no significant differences for VviMPK3 or VviMPK6 (Figure 5). This is not too surprising since AtMKK9 activates AtMPK3 and AtMPK6 by phosphorylation [52]. In addition, the transcript abundance of AtERF6 in Arabidopsis increases with ROS, SA, cold [53], pathogens [53,54], and water deficit [55]. There were no visible signs of pathogen infection in these berries.

**Figure 6:**
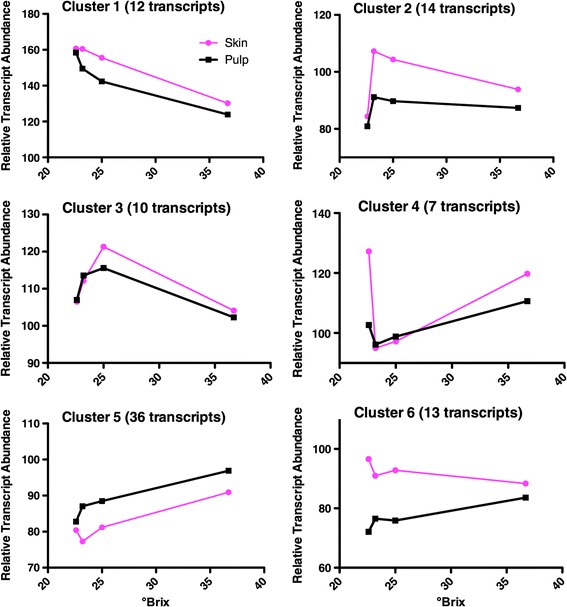
**Average profiles of the transcripts in the 6 clusters of the** ***Vitis vinifera*** **AP2/ERF transcription factor superfamily.**

Additional circumstantial evidence for ethylene signaling in the late stages of berry ripening was that the transcript abundance of many VviERF TFs was significantly affected (92 out of the 130 member AP2/ERF superfamily) by berry ripening (°Brix levels) and/or tissue (Additional file 8). The transcript abundance of 129 members from the berries was determined to be above background noise levels on the microarray (Additional file 8). The expression profiles of the 92 significantly affected AP2/ERF superfamily members were separated into six distinct clusters by hierarchical clustering and indicated that this superfamily had a complex response during berry ripening (Figure 6, Additional file 8). The 12 members of Cluster 1 responded similarly in both the skin and pulp, gradually decreasing with increasing °Brix with a large decrease in transcript abundance at the 36.7 °Brix level. Cluster 2 with 14 members, including 8 members of the VviERF6 clade, had much higher transcript abundance in the skin with a sharp peak at 23.2 °Brix. Cluster 3 (10 members) had similar profiles in both the skin and pulp with a peak abundance at 25° Brix. Cluster 4 with 7 members was a near mirror image of cluster 2, with a sharp valley for transcript abundance in the skin between 23 and 25 °Brix. Cluster 5 had 36 members with a steady increase in transcript abundance in the pulp but no substantial increase in the skin until 36.7 °Brix. Finally, in Cluster 6, there were 13 members with a higher transcript abundance in skins compared to pulp. Their transcript abundance increased with increasing °Brix level, but decreased in the skin.

**Figure 7:**
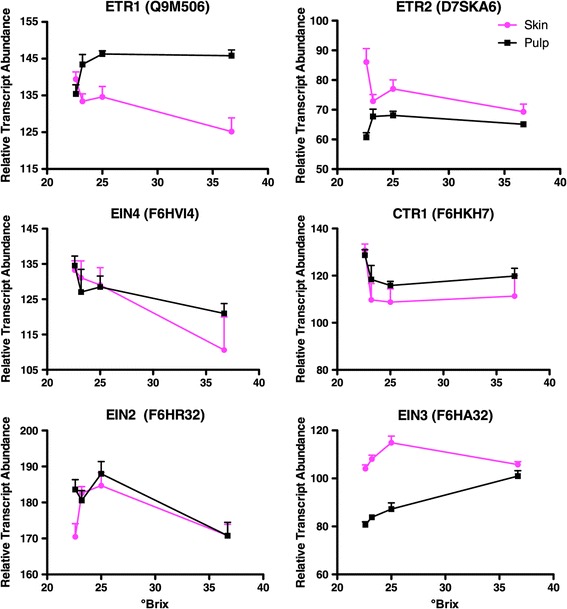
**The transcript abundance of key components of the ethylene signaling pathway.** Data are means ± SE; n = 6.

The transcript abundance of important components of the ethylene signaling pathway characterized in Arabidopsis and presumed to be functional in grape were also affected by °Brix level and tissue (Figure 7). Three different ethylene receptors, VviETR1, VviETR2, and VviEIN4 decreased with °Brix level in the skin, however there was very little or no change in the pulp. Likewise, VviCTR1, another negative regulator of ethylene signaling that interacts with the ethylene receptors, decreased between 22.6 and 23.2 °Brix in both the skin and the pulp. The transcript abundance of the positive regulator, VviEIN2, peaked at 25 °Brix in both the skin and the pulp. AtEIN2 is negatively regulated by AtCTR1 and when it is released from repression, turns on AtEIN3 (a TF) and the ethylene signaling pathway downstream [56]. The transcript abundance of VviEIN3 increased with °Brix level, peaking at 25 °Brix in the skin, and was much higher than in the pulp. Although more subtle, its profile was very similar to VviERF6L1. Derepression of the negative regulators and the increase in positive regulators indicated that ethylene signaling was stimulated during this late stage of berry ripening.

### Flavor pathways

**Figure 8:**
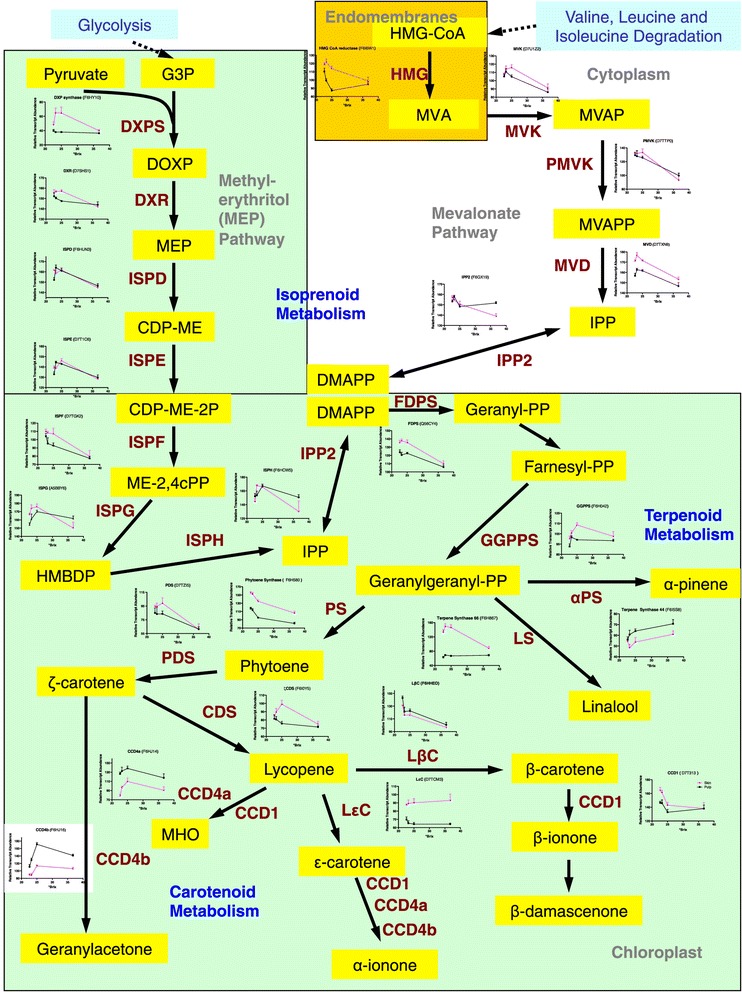
**Expression profiles of transcripts involved in the isoprenoid, carotenoid and terpenoid pathways.** Data are means ± SE; n = 6.

The transcript abundance of many of the genes involved in the isoprenoid biosynthesis pathway peaked between 23 and 25 °Brix level, particularly in the skin; this stimulation of transcript abundance continued in both the carotenoid and terpenoid biosynthesis pathways (Figure 8). DXP synthase is a key regulatory step in isoprenoid biosynthesis and its profile was similar to VviERF6L1; its transcript abundance was correlated with the transcript abundance of several terpene synthases in the terpenoid biosynthesis pathway (Figure 8; Cluster 8 in Additional file 7).

About 50% of the putative 69 functional terpene synthases in the Pinot Noir reference genome have been functionally characterized [12]. Another 20 genes may be functional but need further functional validation or checking for sequencing and assembly errors. On the NimbleGen Grape Whole-Genome array there are 110 probe sets representing transcripts of functional, partial and psuedo terpene synthases in Pinot Noir (Additional file 11). It is uncertain how many may be functional in Cabernet Sauvignon. There were 34 probe sets that significantly changed with °Brix or the °Brix and Tissue interaction effect; 20 of these are considered functional genes in Pinot Noir. Terpene synthases are separated into 4 subfamilies in the Pinot Noir reference genome; they use a variety of substrates and produce a variety of terpenes [12]. Many of these enzymes produce more than one terpene. The top 8 transcripts that peaked in the skin at the 23.2 to 25 °Brix stages were also much higher in the skin relative to pulp (Additional file 11). Five of the eight probesets match four functionally-classified genes in Pinot Noir (VviTPS 55, 60, 64 and 66); these terpene synthases clustered very closely with VviTPS54, a functionally annotated (3S)- Linalool/(E)- Nerolidol synthase [12]. VviTPS58, a (*E,E*)-geranyl linalool synthase, was also in the cluster. The other two probesets match partial terpene synthase sequences in the Pinot Noir reference genome.

The transcript abundance of genes involved with carotenoid metabolism also changed at different °Brix levels and with tissue type (Figure 8). CCDs are carotenoid cleavage dioxgenases and are involved in norisoprenoid biosynthesis. The transcript abundance of VviCCD1 changed signficantly with °Brix level and was higher in skin than pulp, except at 36.7 °Brix. Likewise, the transcript abundance of VviCCD4a and VviCCD4b changed signficantly with °Brix level, but was higher in the pulp than the skin. The transcript abundance of VviCCD4c significantly increased with °Brix level, but there were no significant differences between tissues. VviCCD1 and VviCCD4 produce β- and α-ionone (rose aromas), geranylacetone (floral rose aroma), and 6-methyl-5-hepten-2-one (MHO; ether odor and fragrance) in grapes [57,58]. There were no significant effects on the transcript abundance of VviCCD7. The transcript abundance of VviCCD8 significantly increased with °Brix level and was higher in pulp than skin. Phytoene synthase, which was also increased in the skin compared to the pulp (Figure 8), and VviCCD1, have been associated with β-ionone and β-damascenone biosynthesis [59].

**Figure 9:**
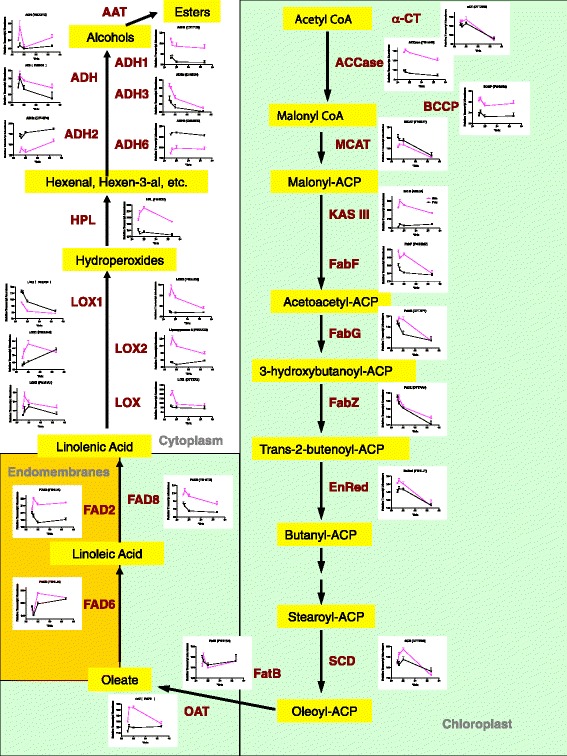
**Expression profilies of transcripts involved in fatty acid metabolism.** Data are means ± SE; n = 6.

Other important grape flavors are derived from the fatty acid metabolism pathway and lead to the production of aromatic alcohols (e.g. hexenol and benzyl alcohol) and esters. The transcript abundance of many genes associated with fatty acid biosynthesis and catabolism changed with °Brix level (Figure 9). In particular the transcript abundance of a number of genes were correlated with the transcript abundance of VviERF6L1 including VviACCase, Acetyl-CoA carboxylase; KAS III (3-ketoacyl-acyl carrier protein synthase III); VviOAT, (oleoyl-acyl carrier protein thioesterase); VviFAD8; (fatty acid desaturase 8); VviLOX2 (lipoxygenase 2) and VviHPL (hydroperoxide lyase). The transcript abundance of alcohol dehydrogenases (ADHs) was affected by tissue and °Brix level (Figure 9). Some ADHs are associated with the production of hexenol and benzyl alcohol [59].

**Figure 10:**
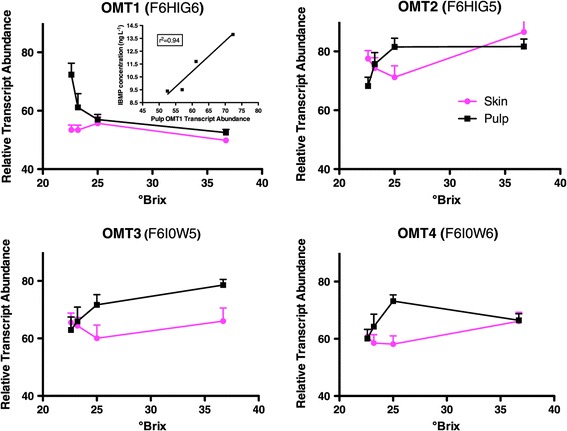
**The transcript abundance of four O-methyltransferases (OMTs) in Cabernet Sauvignon berries.** Inset: the correlation of OMT1 transcript abundance in the pulp with 2-isobutyl-3-methoxypyrazine (IBMP) concentrations in the berries. Data are means ± SE; n = 6.

Methoxypyrazines give herbaceous/bell pepper aromas [19]. They are synthesized early in berry development and gradually diminish to very low levels at maturity. Nevertheless, humans can detect very low concentrations of these aroma compounds. Four enzymes, VviOMT1, VviOMT2, VviOMT3 and VviOMT4 (O-methyltransferases), synthesize methoxypyrazines [19,60,61]. The transcript abundance of VviOMT1 was higher in the pulp than the skin (Figure 10). In addition, the transcript abundance of VviOMT1 decreased significantly with °Brix level in the pulp. There were no significant differences in the trancript abundance in the skin or pulp for VviOMT2, VviOMT3 or VviOMT4 (Figure 10). There was a high correlation (r = 0.97) of the transcript abundance of VviOMT1 in the pulp (but not the skin) with 2-isobutyl-3-methoxypyrazine (IBMP) concentrations in the berries (Figure 10). The transcript abundance of VviOMT2, VviOMT3, or VviOMT4 in either skin or pulp was not correlated with IBMP concentrations (data not shown). This is consistent with the suggestion that the pulp is the main contributor of IBMP in the berry [62]. Our data indicated that VviOMT1 in the pulp may contribute to the IBMP concentration in these berries.

### Other fruit ripening TFs

Orthologs of RIN and SPL (squamosa) tomato transcription factors, which are known to be very important fruit ripening trancription factors [63,64], were at much higher transcript levels in the skin and decline with °Brix level (Additional file 2). The transcript abundance of the VviNOR ortholog in grape was higher in the pulp and increased slightly to peak at 25 °Brix. In addition, the transcript abundance of VviRAP2.3, an inhibitor of ripening in tomato (called ERF6 in tomato), decreased in the skin with a valley at 23.2 °Brix; it belongs to Cluster 4 of the AP2/ERF superfamily (Figure 6, Additional file 8).

Of particular interest was VviWRKY53 [UniProt: F6I6B1], which had a very similar transcript profile as VviERF6L1 (data not shown). AtWRKY53 is a TF that promotes leaf senescence and is induced by hydrogen peroxide [65,66]. This is the first report we know of implicating WRKY53 in fruit ripening (senescence). AtERF4 induces AtWRKY53 and leaf senescence [67], so the interactions between WRKY and ERF TFs are complex. WRKY TFs bind to the WBOX elements in promoters and VviERF6L1 has a number of WBOX elements in its promoter (data not shown). In addition, AtMEKK1 regulates AtWRKY53 [68] and the transcript abundance of VviMEKK1 peaked at 23.2 °Brix in the skin as well. Interestingly, the transcript abundance of both VviERF4 and VviERF8, whose orthologs in Arabidopsis promote leaf senescence, were at their highest level of transcript abundance at the lowest °Brix levels (earliest stages) examined in this study (Cluster 1 in Additional file 8).

## Discussion

This study focused on the very late stages of the mature Cabernet Sauvignon berry when fruit flavors are known to develop. Cabernet Sauvignon is an important red wine cultivar, originating from the Bordeaux region of France. It is now grown in many countries. Wines made from Cabernet Sauvignon are dark red with flavors of dark fruit and berries (i.e. blackcurrants, blueberry, blackberry and raspberry) [69,70]. They also can contain herbaceous characters such as green bell pepper flavor (IBMP) that are particulary prevalent in underripe grapes [3,43,69,70].

Grape flavor is complex consisting not only of many different fruit descriptors, but descriptors that are frequently made up of a complex mixture of aromatic compounds [3]. For example, black currant flavor, in part, can be attributed to 1,8-cineole, 3-methyl-1-butanol, ethyl hexanoate, 2-methoxy-3-isopropylpyrazine, linalool, 4-terpineol, and β-damascenone [71-73] and major components of raspberry flavor can be attributed to α- and β-ionone, α- and β-phellandrene, linalool, β-damascenone, geraniol, nerol and raspberry ketone [74,75]. Some common volatile compounds found in the aroma profiles of these dark fruits and berries include benzaldehyde, 1-hexanol, 2-heptanol, hexyl acetate, β-ionone, β-damascenone, linalool, and α-pinene [13,71,76,77].

In a study of Cabernet Sauvignon grapes and wines in Australia, Cabernet Sauvignon berry aromas were associated with trans-geraniol and 2-pentyl furan and Cabernet Sauvignon flavor was associated with 3-hexenol, 2-heptanol, heptadienol and octanal [70]. In another comprehensive study of 350 volatiles of Cabernet Sauvignon wines from all over Australia, the factors influencing sensory attributes were found to be complex [78]; in part, norisoprenoids and δ − and γ-lactones were associated with sweet and fruity characteristics and red berry and dried fruit aromas were correlated with ethyl and acetate esters. In Cabernet Sauvignon wines from the USA, sensory attributes were complex also and significantly affected by alcohol level of the wine [69]. Linalool and hexyl acetate were postitively associated with berry aroma and IBMP was positively correlated with green bell pepper aroma. In France, β-damascenone was found to contribute to Cabernet Sauvignon wine aroma [79].

Thus, flavor development in berries and wines is very complex, being affected by a large number of factors including genetics, chemistry, time and environment. In this paper we begin to examine the changes in transcript abundance that may contribute to flavor development. We show that the transcript abundance of many genes involved in fatty acid, carotenoid, isoprenoid and terpenoid metabolism was increased in the skin and peaked at the °Brix levels known to have the highest fruit flavors (see Figures 8 and 9). Many of these are involved in the production of dark fruit flavors such as linalool synthases, carotenoid dioxygenases and lipoxygenases. These genes serve as good candidates for berry development and flavor markers during ripening. A broader range of studies from different cultivars, locations and environments are needed to determine a common set of genes involved in berry and flavor development.

A similar study was conducted on the production of volatile aromas in Cabernet Sauvignon berries across many developmental stages [13], including a detailed analysis of the °Brix levels that was surveyed in this study. They found that the production of alcohol volatiles from the lipoxygenase pathway dominated in the later stages of berry ripening and suggested that the activity of alcohol dehydrogenases also could play an important role.

The abundance of the transcript of VviOMT1 decreased in the pulp with increasing °Brix level and was correlated with IBMP concentrations in the late stages of berry development in this study. Both OMT1 [19] and OMT3 [60,61] have been shown to synthesize IBMP. Furthermore, the transcript abundance of each gene has been correlated with IBMP concentration, but the transcript abundance of each gene cannot fully account for the total IBMP present in all genotypes and conditions [19,60-62]. OMT3 was found to be the major genetic determinant for this trait in two independent studies [60,61]. Nevertheless, it is possible that OMT1 may contribute to the IBMP concentration, because OMT1 can synthesize IBMP [19] and it is located at the edge of a QTL significantly contributing to this trait [60]. Furthermore, the majority of IBHP (2-isobutyl-3-hydroxypyrazine), the precursor for the OMT1 and OMT3 biosynthesis of IBMP, is produced in the pulp of the berry [62] complicating the factors that influence IBMP concentration. Our results raise questions that require additional research to clarify this relationship of transcript abundance to IBMP concentration, including determination of the rates of biosynthesis and catabolism, enzyme activities, volatilization of IBMP from the berry, as well as the concentrations of substrates for the enzymes involved [62].

There are a number of other transcriptomic ripening studies in grapes and other fruit species [24,80-86]. Many of these have compared broad developmental stages with partial (not whole) genome microarrays. One study compared transcriptomic responses of the lates stages of ripening of whole berries of Chardonnay [86]. This study used a different microarray platform with only about half of the genome represented on the array. In this study, 12 genes were found to be differentially expressed in each of the 3 different stages investigated. There were approximately another 50 genes that were differentially expressed at one stage versus another. Several genes were proposed as good candidates for markers of ripeness and these were also examined in Cabernet Sauvignon berries using qPCR. Several of these candidate genes are consistent with our results in the present study. They include CCD4a (F6HJ14), a late embryogenesis abundant protein (F6HKF4), a dirigent-like protein (F6GUG1), and an S-adenosyl-L-methionine:salicylic acid carboxyl methyltransferase (SAMT; F6GWU1). Of these, the transcript expression of SAMT was found to be temperature insensitive [86].

Like the previous study, the present study focused on very close stages in the mature berry when fruit flavors are known to develop. In contrast to the previous study on Chardonnay [86], there were massive changes in the transcript abundance in hundreds of GO categories over this narrow window of ripening. This may in part be due to using six biological replicates rather than the standard three, which probably improved the detection of significantly changing transcripts. In addition, we used a different threshold level for statistical signficance and an improved microarray platform, which was able to detect double the number of transcripts.

In the present study, many differences were found between the skin and the pulp, °Brix levels and the interaction of tissue and °Brix. Important fruit ripening processes were affected including ethylene signaling, senescence, volatile aroma production, lipid metabolism and cell wall softening. These data indicate that fruit ripening in the late stages of maturity is a very dynamic and active process.

### Importance of ethylene in fruit ripening and senescence: climacteric and nonclimacteric fruit

Ethylene is involved in climacteric fruit ripening with a CO_2_ burst preceding the rise in ethylene [64]. In tomato, this occurs at the time the seeds become mature in the mature green fruit stage. At this stage, tomato fruits become sensitive to ethyene and can continue through the ripening stage. Prior to the mature breaker stage, ethylene cannot promote tomato ripening to full ripeness.

In nonclimacteric fruit, there is no respiratory burst of CO_2_ and the ripening of most nonclimacteric fruits was thought not to respond significantly to an extra application of ethylene [64]. However, recently some nonclimacteric fruit such as strawberry [87], bell pepper [88] and grape [33] have been found to produce a small amount of ethylene and appear to have responses to ethylene at certain stages. In the study of grapes, this peak was observed just before the start of veraison, followed by decreases in ethylene concentrations for several weeks afterwards; the late mature stages of ripening were not examined.

Ethylene action is dependent upon ethylene concentration and ethylene sensitivity or signaling [89]. In this study, there were clear and signficant changes in transcript abundance of genes involved in ethylene signaling and biosynthesis in the late stages of berry ripening. Seeds become fully mature at this time (based upon our observations that green seeds turn brown at this stage). Perhaps there is a signal from the seeds when they become mature that allows the fruit to ripen and senesce? Perhaps small amounts of ethylene are produced or there is a change in sensitivity to ethylene?

Seymour et al. [63] suggested the response of EIN3 might be a common signaling mechanism for both climacteric and nonclimacteric fruit. The responses of VviEIN3 in this study and in a pepper fruit ripening study [90] are consistent with this hypothesis. In addition, the transcript abundance of VviEIN3 in grape is very responsive to ethylene and the ethylene inhibitor, MCP [91].

There are many other factors other than fruit development that can influence ethylene signaling. Could chilling of the fruit or other aspects of the processing of the grapes influence these responses? Could there be some influence of other abiotic or biotic stresses? These are questions that can only be addressed in future studies with additional experiments that are designed to answer these questions.

## Conclusions

In summary, there are dynamic metabolic changes in the late stages of berry ripening including large changes in abundance of transcripts involved in ethylene signaling, fruit softening, terpenoid biosynthesis, fatty acid metabolism and amino acid metabolism. Many of these changes can have important effects that may result in the production of volatile aromas that influence berry flavor. A unique clade of the subfamily IX ERF transcription factors was highly expressed in the skin during this ripening phase. The important implications of this research is that ethylene may play a bigger role in this nonclimacteric fruit than previously thought, particularly in the late stages of ripening when flavors are produced.

## Methods

### Plant material and experimental conditions

#### Commercial vineyard and row randomization plan

A vineyard from a commercial winery in Paso Robles CA, planted with *Vitis vinifera* L. cv. Cabernet Sauvignon (clone 8 scion on 1103 Paulsen rootstock) was dedicated to the study. Thirty-three vineyard rows, established as three separate blocks of 11 consecutive rows, were assigned to the project based on a completely randomized block design. The row orientation of the vineyard was north–south.

#### Fruit samples

IBMP concentrations of berries during maturation were determined according to the method of Koch [92]. Twenty bunches per row were picked at each harvest. Samples were collected from six assigned rows at each harvest date in the first five harvest dates and from three assigned rows in the last harvest. Two clusters per vine were sampled from the west arm on the cordon every ten plants. Clusters were carried in a cooler with dry ice to the Heymann laboratory and stored at −80°C until analysis. Samples for transcriptomic analyses were packed on ice and shipped overnight to the Cramer laboratory before being frozen in liquid nitrogen and stored at −80°C. This shipping procedure of the grapes was deemed to be similar to grape processing in a winery, where grapes sit in bins or coolers before being pressed.

### Chemical analyses of fresh and previously frozen grapes

Titratable acidity and pH were determined using autotritation (Mettler Toledo DL50 autotitrator and 60 Auto sampler with LABX software; Columbus, Ohio). Total soluble solids (sugar being the most signficant component) content of grape juices was determined at harvest with a hydrometer. pH and titratable acidity were determined immediately after harvest.

#### IBMP analysis and grape sample preparation

Thirty-six grams of berries were used for each sample to be analyzed by Head Space-Solid Phase MicroExtraction-Gas chromatography–mass spectrometry (HS-SPME-GC-MS). Frozen whole berries (36 g) were thawed and placed into a 50 mL plastic Falcon™ tube. A 10 mL solution of 2 mM NaF prepared with MilliQ water, containing 50 ng of deuterated IBMP ([^2^H3]IBMP, CDN Isotopes, Quebec, Canada, 98.2% pure) and four different concentrations of standard IBMP (Methoxypyrazine Specialties, Atlanta, GA, 99% pure by GC-MS) ranging between 0.0 ng L^−1^ and 50 ng L^−1^, was added to the berries in the tube. The solution was homogenized for 45 s and centrifuged (5000 rpm for 5 min). The pellet was discarded. Three 20 mL glass round bottom dark headspace vials were labeled for each instrumental sample replication and three grams of NaCl were added to each vial. Ten mL of the supernatant following centrifugation were transferred into each of the three vials and the caps closed tightly and equilibrated for 1 h at room temperature prior to HS-SPME analysis. Six field replicates were analyzed with two instrumental replicates.

#### HS-SPME-GC-MS analysis

The basic conditions of Chapman et al. [93] were used for all analyses. Extractions were performed using a 23 gauge, 2 cm divinylbenzene/Carboxen™/polydimethylsiloxane (PDMS/DVB/CARB) SPME fiber (Supelco, Bellafonte, PA), that was conditioned and cleaned according to manufacturer’s specifications. The SPME fiber was placed in the headspace of each sample vial and the sample extracted for 30 min at 40°C with continuous stirring. The SPME fiber was removed from the vial and placed into the gas chromatography-mass spectrometer (GC-MS) inlet which was equipped with a 0.7 mm straight glass liner. An Agilent 6890 GC with a 5973MSD (Agilent, Santa Clara, CA) and Gerstel MPS2 autosampler (Gerstel, Inc., Columbia, MD) were used for all analyses. The injector was held at 260°C with no purge for 5 min for the analytes to desorb from the fiber. The purge was switched to 50 mL min^−1^ with the fiber in the inlet for an additional 5 min (no carry over was detected).

An HP 5MS capillary column (30 m × 0.25 mm ID, 0.25 μm film thickness; Agilent) was used for separation. The oven temperature was maintained at a constant temperature of 40°C for 5 min, then increased 2.5°C min^−1^ to 80°C, 5°C min^−1^ to 110°C, and 25°C to 230°C before holding steady for 5 min. The MSD interface was held at 280°C and the carrier gas was Helium at a constant pressure of 4.77 psi with a nominal initial flow of 0.8 mL min^−1^ and average linear velocity of 32 cm sec^−1^. Selected ion monitoring was used (according to [93]) at mass channels of *m/z* = 124 and 94 for IBMP and *m/z* = 127 and 154 for deuterated IBMP. Peak areas of the ions *m/z* 124 and 127 were used for quantification and *m/z* 94 and 154 were used as qualifiers. Retention times for [2H3]IBMP and IBMP were 26.75 min and 26.83, respectively.

#### Calibration and quantification

IBMP (99% pure) was purchased from Methoxypyrazine Specialties (Atlanta, GA). Standard IBMP calibration samples were prepared (according to the method by [93]) in 10 mL of model wine (2 g L^−1^ of potassium bitartrate, 12% ethanol), for wine analysis, to give IBMP concentrations of 0, 0.1, 0.5, 1, 2, 5, 15, 30, and 50 ng L^−1^. A 10 mL aliquot of each standard solution was transferred into a 20 mL headspace vial that contained 3 g of NaCl. The internal standard (IS), [2H3]IBMP, was also added to each vial at a final concentration of 50 ng L^−1^. The standards were prepared in triplicate and were analyzed by HS-SPME-GC-MS as described above. Peak area ratios of IBMP and [2H3]IBMP were used to create linear calibration curves.

IBMP concentrations in grape homogenates were determined by the method of standard addition Koch [92]. After grape sample preparation and extraction, the supernatant was analyzed by HS-SPME-GC-MS as described for the wines. The peak area ratio of IBMP relative to [2H3]IBMP was used to create a standard addition calibration curve for each sample. The concentration of IBMP in the supernatant was calculated from the linear regression equation of the calibration curve at the point where the y-intercept is equal to zero. IBMP concentrations in the original fruit were calculated from the supernatant concentration by correcting for the dilution of the original 36 g of grape sample with 10 mL of aqueous [2H3]IBMP/IBMP solution and assuming a density of 1.0 g/mL for the homogenate solution. Corrected IBMP concentrations in grape were expressed as ng L^−1^ of grape juice extract.

### RNA extraction

Skins were peeled from frozen berries with a razor blade for all berries on the clusters of each biological replicate. Separated skins and pulp of all the berries of each biological replicate were pooled (without seeds) and ground in a frozen mortar and pestle. Total RNA was extracted from the finely ground skins and pulp in liquid nitrogen using Qiagen RNeasy Plant MidiKit columns as previously described [94]. The total RNA was further purified using a Qiagen RNeasy Plant Mini Kit according to the manufacturers’ instructions. RNA quality and quantity were determined using a Nanodrop 2000 spectrophotometer (Thermo Scientific) and a Bioanalyzer Chip RNA 7500 series II (Agilent). For samples characterized by ratio of 260/230 < 1.8, the total RNA was precipitated with LiCl to remove contaminants. LiCl was mixed with total RNA to a final concentration of 2.5 M, incubated overnight at 4°C, and centrifuged at 13,000 g, and the pellet was washed with 70% ethanol before resuspending in water.

### Microarray hybridization and data extraction

We performed cDNA synthesis, labeling, hybridization, and washing steps according to the NimbleGen Arrays User’s Guide (version 3.2). Ten μg of total RNA was used for each sample. Labeled cDNA was hybridized to a NimbleGen microarray 090818 Vitis exp HX12 (Roche, NimbleGen Inc., Madison, WI, USA), which contains probes targeted to 29,549 grapevine genes predicted from the V1 annotation of the 12x grapevine genome (https://urgi.versailles.inra.fr/Species/Vitis/Annotations), and 19,091 random probes as negative controls. Each microarray was scanned using an Axon GenePix 4400A (Molecular Devices, Sunnyvale, CA, USA) at 532 nm (Cy3 absorption peak) and GenePix Pro7 software (Molecular Devices) according to the manufacturer’s instructions. All microarray expression data will be made available in the Gene Expression Omnibus (GEO) website under the accession name GSE55302 on November 1, 2014.

### Phylogenetic tree construction

The gene models for AP2/ERF transcription factor superfamily from *Arabidopsis thaliana* (TAIR10) and *Vitis vinifera* (V1) were retrieved from Gramene (40) by searching for the AP2 domain (SM00380). The sequences were aligned with MUSCLE [95,96] using their amino acid sequences with the default settings for the MUSCLE software. The phylogenetic tree was constructed by MUSCLE and was imported into the FigTree (http://tree.bio.ed.ac.uk/software/figtree/) application for further modifications and annotation of the appearance of the phylogenetic tree.

### Data analysis and normalization

All NimbleGen custom oligonucleotide array images were first examined visually in their raw data format for gross spatial variation due to fibers or bubbles. Two arrays contained considerable spatial variation due to a possible fingerprint or smear. Rather than excluding the entire arrays, a strict quality control process on the individual expression values within and across the regions of question was performed. These are the same protocols followed for one of our previous studies performed on a custom NimbleGen array of similar type [97].

All raw array data were processed and normalized first by Robust Multi-Array Average (RMA) [98] using the R package affy [99]. Specifically, expression values were computed by applying the RMA model of probe-specific correction of perfect match probes. The processed probe values were then normalized via quantile normalization, and a median polish was applied to compute one expression measure from all probe values.

The six expression measurements for each biological state were inspected individually for each element on the array. Of the 236,400 sets of sextuplets, 11.6% exhibited a coefficient of variation greater than 50%. Any set of sextuplets that displayed a coefficient of variation of greater than 0.5, and that included one or more replicated measures lying more than 1.35 standard deviations way from the mean was scrutinized. The maximum standard deviation for six replicates in this dataset was 2.04; 15% of all measurements observed were greater than 1.35 standard deviations away from the mean of their set of replicates. If one of the six replicates was more than 1.35 standard deviations away from the mean across the six replicates, this indicated a very high probability (87%) that the remaining five measures were very similar, and that this replicate was an outlier. Of the 1,418,400 expression values measured in this experiment, 4489 (0.32%) were excluded as single outliers, and 1586 (0.11%) were excluded as a set of two outlying values in a set of replicates. Upon this correction, there remained 429 sets of sextuplets that still had coefficients of variation that were still greater than 0.85, which were completely excluded from further analysis. The remaining 1,393,564 (98.2%) values had an average coefficient of variation of 0.264, which is typical of the microarray experiments processed by the UNR Center for Bioinformatics. We found that these thresholds allowed us to identify gross outlying individual measurements within a quadruplicate set [97,100].

A simple 2-way ANOVA was performed on the normalized processed data to determine which features on the array were differentially expressed across the °Brix levels, the tissues, and the interaction between these two effects.

The processed and normalized expression values were not normally distributed, but moderate deviations from normality have been found to have little effect on ANOVA F-tests [101]. A multiple testing correction was applied to the p-values of the ANOVA [44], and any feature with a significant interaction term with adjusted p-value < 0.05 was examined further.

The Pearson correlation coefficient of the linear regression of 10 pairs of microarray/qRTPCR log2 (CAM/C3) expression ratios was computed as 0.942.

Using Fisher’s Z-transform, the confidence interval of the coefficient was computed, with a p-value < <0.001.

### Evaluation of NimbleGen microarray probe specificity

NimbleGen microarray probes were aligned against the database of V1 annotation of the 12x grapevine genome (https://urgi.versailles.inra.fr/Species/Vitis/Annotations) using the BLASTn algorithm with the following parameters: −S 1 -F F -W 7 -v 5 -b 10000 -e 0.0 [102]. Alignments with more than 80% identity and coverage were retained as putative. Hits whose predicted melting temperature (Tm) was higher than the minimum Tm of perfectly matching probes minus 10°C were considered as putative cross-hybridizations [103,104].

### Validation of the NimbleGen microarray by qPCR

qPCR was performed on 12 genes in a previous publication [105].

The qPCRexpression levels of the twelve genes was compared to the normalized (RMA) microarry expression values across the conditions under which each gene was measured. While nine of the twelve genes showed a Pearson correlation coefficient of greater than 0.82, the expression levels of three genes were not in agreement between the two methods (correlation was less than 0.65).

The average correlation between the twelve genes’ qPCR and microarray values was 0.80; the average correlation between the nine well-correlated genes was 0.95.

Based on two other experiments performed in the Cramer lab, we found an ERF gene to have a Pearson correlation coefficient of 0.67 across 24 independent measurements, and a MYB gene to have a Pearson correlation of 0.91 across the same 24 measurements. Thus, approximately 71% of all genes tested by qPCR were correlated at or above 0.80.

### Gene set enrichment analysis

Overrepresentation of functional groups in gene sets were determined using the BinGO [45] application within Cytoscape [106]. The latest GO annotations of the V1 genes were downloaded from Ensembl Plants (plants.ensembl.org) on December 20, 2013. Overrepresentation was determined using the Nimblegen whole genome array based on the V1 assembly as a reference and at the adjusted p-value < 0.05 using the Benjamini & Hochberg False Discovery Rate correction in BinGO.

### Gene clustering

Significantly changing transcripts with similar abundance profiles were grouped using hierarchical clustering (HCl) and k-means clustering algorithms in the MeV software package [107]. As expression data were not normally distributed, the non-parametric Spearman Correlation Coefficient distance metric was used in the clustering procedure. The approximate number of clusters to use in the k-means clustering was estimated with the Figure of Merit algorithm in the MeV software package. The actual number of clusters was fine-tuned by examining the groups of transcript abundance profiles with smaller and larger clusters for the best and most accurate representation of the cluster profile in each cluster. Other settings used during the clustering process were a maximum of 50 iterations for the clustering and clustering by gene functions. A final evaluation of the clusters was then made to determine which group of clusters represented the data more accurately. HCl produced good clusters for AP2/ERF TF superfamily but not for the set of significant transcripts for the °Brix x Tissue interaction. For the latter group, the k-means clustering was determined to be a better representation of the profiles because there was a tighter group of correlating genes in the clusters.

## Additional files

Additional file 1
**Details of the various berry quality parameters measured.**
Additional file 2
**Annotation, transcript abundance values, and statistics of all genes on the Nimblegen Grape Whole-Genome microarray for the pulp and skin of Cabernet Sauvignon berries at different °Brix levels.**
Additional file 3
**BinGO analysis of all significant transcripts changing with °Brix.**
Additional file 4
**Overrepresented GO categories of all significant transcripts higher in skin than pulp at 23 °Brix.**
Additional file 5
**Overrepresented GO categories of all significant transcripts higher in pulp than skin at 23 °Brix.**
Additional file 6
**Overrepresented GO categories of all significant transcripts changing with the °Brix x Tissue interaction.**
Additional file 7
**Individual transcript members of each cluster and their overrepresented GO categories for all significant transcripts within each of the 10 clusters in the °Brix x Tissue interaction.**
Additional file 8
**Annotation information and expression data for the AP2/ERF transcription factor superfamily.**
Additional file 9
**Phylogenetic tree of the AP2/ERF trancrpition factor superfamilies of** ***Vitis vinifera*** **and** ***Arabidopsis thaliana*** **.** The abbreviations, RAV and AP2 refer to the subfamilies. Roman numerals refer to ERF subfamilies. Colors are linked to subfamilies and described in Additional file 8. Additional file 10
**Expression profiles of the 12 members of the clade of VviERF6 transcription factors.**
Additional file 11
**Annotation and expression data for the terpene synthase family on the NimbleGen array.** Yellow highlights indicate transcripts significantly changing below an adjusted false discovery rate of 0.05 for the °Brix x Tissue interaction term; red highlights indicate transcripts significantly changing below an adjusted false discovery rate of 0.05 for °Brix.
