# Supplementary figures and images for: Transcriptomic analysis of the late stages of grapevine (Vitis vinifera cv. Cabernet Sauvignon) berry ripening reveals significant induction of ethylene signaling and flavor pathways in the skin
Source: BMC Plant Biol. 2014 Dec 19;14:370. doi: 10.1186/s12870-014-0370-8 (PMC4312598; doi:10.1186/s12870-014-0370-8)

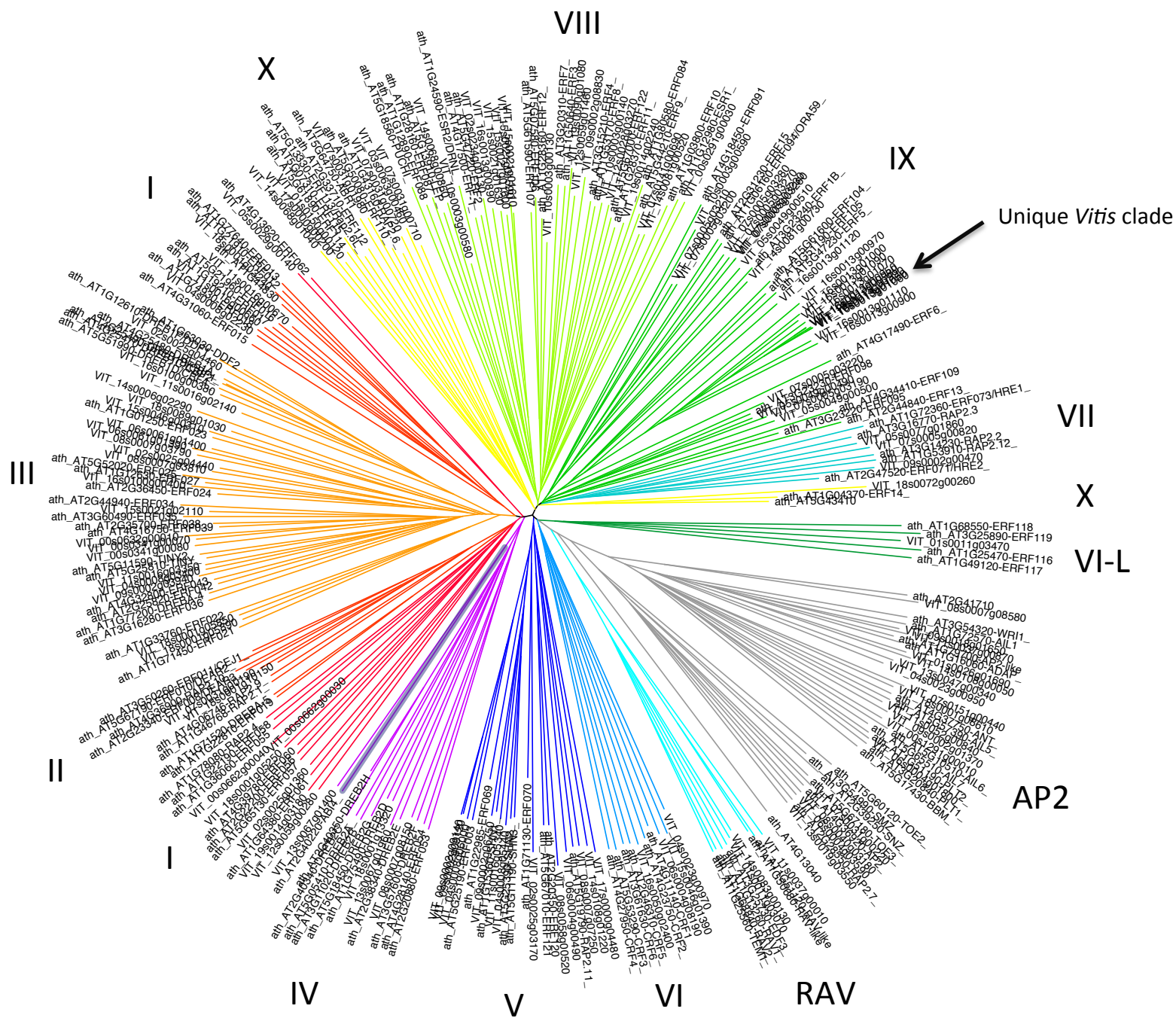

Supplement: Additional file 9 — Phylogenetic tree of the AP2/ERF trancrpition factor superfamilies of Vitis vinifera and Arabidopsis thaliana . The abbreviations, RAV and AP2 refer to the subfamilies. Roman numerals refer to ERF subfamilies. Colors are linked to subfamilies and described in Additional file 8. [file 12870_2014_370_MOESM9_ESM.pdf]

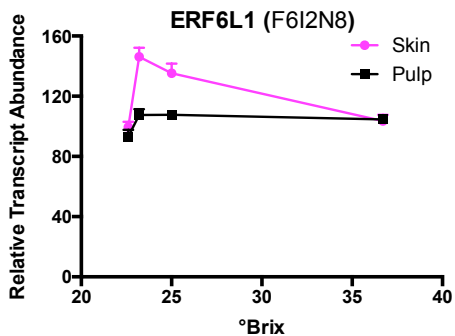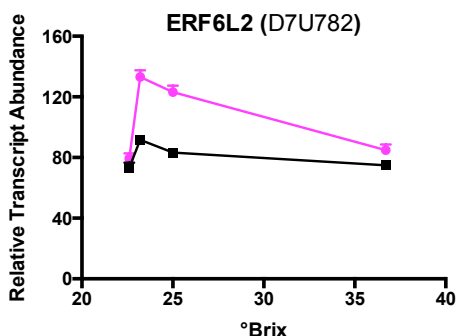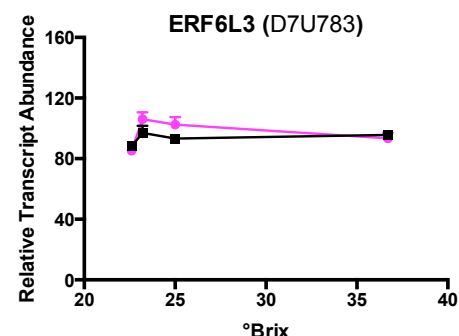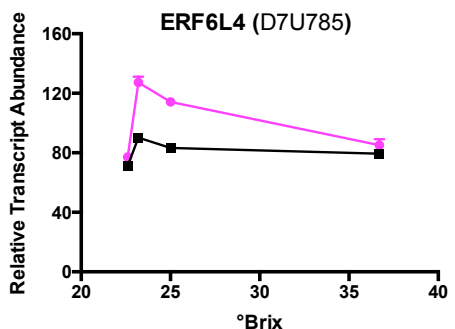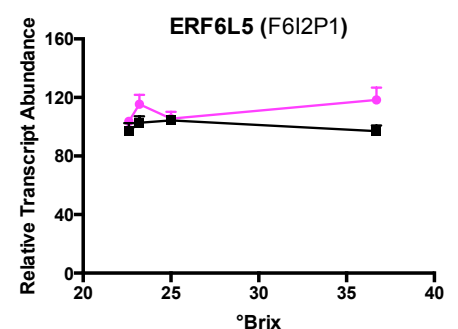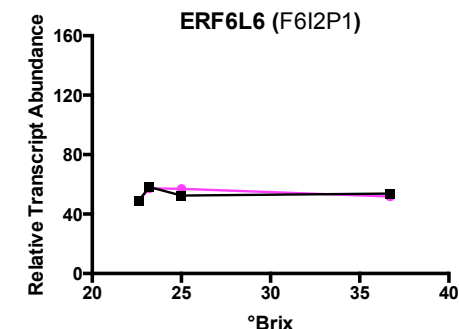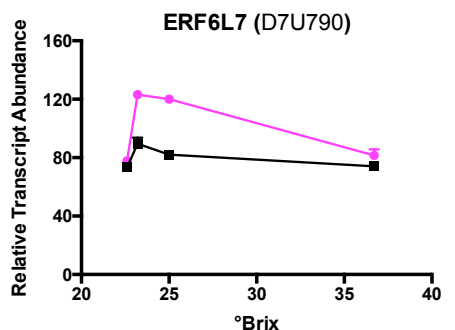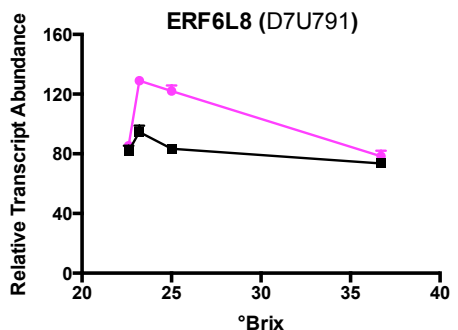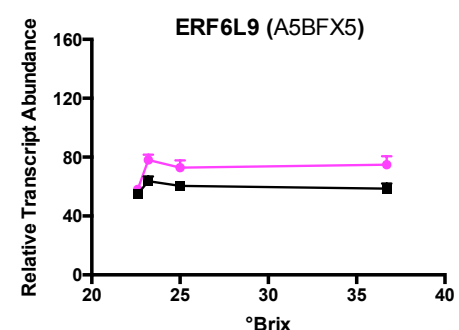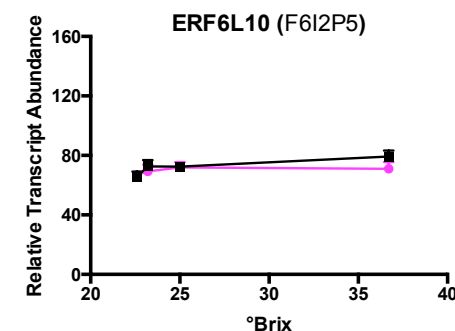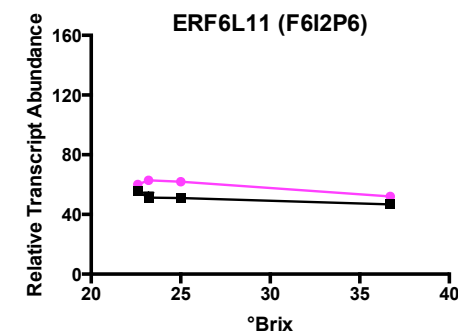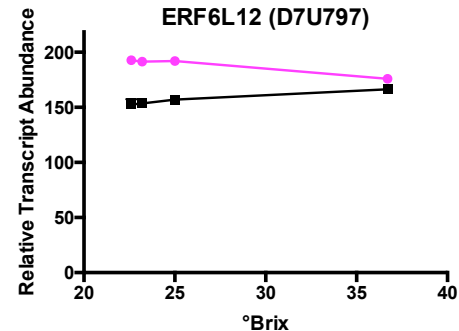

Supplement: Additional file 10 — Expression profiles of the 12 members of the clade of VviERF6 transcription factors. [file 12870_2014_370_MOESM10_ESM.pdf]
